# Observational study of rebiopsy in EGFR-TKI-resistant patients with *EGFR* mutation-positive advanced NSCLC

**DOI:** 10.1038/s41598-022-10288-8

**Published:** 2022-04-16

**Authors:** Kenichi Koyama, Satoru Miura, Satoshi Watanabe, Satoshi Shoji, Jun Koshio, Yoshiki Hayashi, Daisuke Ishikawa, Ko Sato, Takao Miyabayashi, Masaaki Okajima, Takeshi Ota, Tomohiro Tanaka, Naoya Matsumoto, Hideyuki Kuriyama, Tetsuya Abe, Koichiro Nozaki, Kosuke Ichikawa, Rie Kondo, Hiroshi Tanaka, Toshiaki Kikuchi

**Affiliations:** 1 Department of Internal Medicine, Niigata Cancer Center Hospital, 2-15-3, Kawagishi-cho, Chuo-ku, Niigata city, 951-8566 Japan; 2 Department of Respiratory Medicine and Infectious Diseases, Niigata University Graduate School of Medical and Dental Sciences, 1-757 Asahimachidori, Chuouku, Niigata, 951-8510 Japan; 3 Department of Respiratory Medicine, Nagaoka Red Cross Hospital, Niigata, Japan; 4 Department of Respiratory Medicine, Nagaoka Chuo General Hospital, Niigata, Japan; 5 Department of Respiratory Medicine, Niigata Prefectural Central Hospital, Niigata, Japan; 6 Department of Respiratory Medicine, Niigata City General Hospital, Niigata, Japan; 7 Department of Respiratory Medicine, Saiseikai Niigata Hospital, Niigata, Japan; 8 Department of Respiratory Medicine, Shibata Hospital - Niigata Prefectural Hospital, Niigata, Japan; 9 Department of Respiratory Medicine, Nishi Niigata Chuo Hospital, Niigata, Japan; 10 Department of Respiratory Medicine, Sado General Hospital, Sado, Japan; 11 Department of Respiratory Medicine, Tsuruoka Municipal Shonai Hospital, Tsuruoka, Japan; 12 Department of Respiratory Medicine, Shinrakuen Hospital, Niigata, Japan; 13 Department of Respiratory Medicine, Niigata Medical Center, Niigata, Japan

**Keywords:** Cancer, Oncology

## Abstract

The identification of acquired resistance mutations has been essential in non-small-cell lung cancer (NSCLC) patients with *epidermal growth factor receptor (EGFR)* active mutations. Rebiopsy plays a pivotal role in selecting the optimal treatment for patients who develop resistance to initial EGFR-tyrosine kinase inhibitors (EGFR-TKIs). This multicenter, observational study was conducted to investigate the details of rebiopsy in Japanese clinical practice. The primary endpoints were the implementation rate of rebiopsy and the concordance rate for T790M mutation detection between histological and cytological specimens using the cobas EGFR Mutation Test, version 2. One hundred ninety-four patients with *EGFR*-mutant NSCLC were enrolled, and 120 patients developed acquired resistance to EGFR-TKIs. The median age was 68 years (range 20–87), and 52.5% of the patients were women. Rebiopsy was performed in 109 patients, and the implementation rate of rebiopsy was 90.8%. The success rates of rebiopsy in the total, histology, cytology and liquid biopsy populations were 67.9%, 81.3%, 66.7% and 43.8%, respectively. The positive percent agreement and the negative percent agreement in the detection of the T790M mutation between the histological and cytological specimens were both 90.9%. Obtaining histological or cytological tissue samples at rebiopsy may contribute to improving the detection rate of the T790M mutation (trial registration number: UMIN000026019).

## Introduction

Molecular targeted therapies and immunotherapy using immune checkpoint inhibitors have emerged as essential modalities in various cancer treatments. *Epidermal growth factor receptor (EGFR)* mutations were strongly correlated with the clinical benefit of EGFR-tyrosine kinase inhibitors (EGFR-TKIs) and the first druggable driver mutation in non-small-cell lung cancer (NSCLC) ^1,2^. It has been reported that first- or second-generation EGFR-TKIs showed significant clinical benefits compared with standard chemotherapy in patients with *EGFR* active mutations ^3–8^. After the discovery of *EGFR* active mutations, various druggable driver mutations and TKIs were identified in lung adenocarcinoma, such as *EML4-ALK, ROS1*, and *BRAF* mutations. TKIs for these kinds of druggable driver mutations are highly recommended in the current treatment guidelines for NSCLC ^9–11^. In current clinical practice, the detection of druggable somatic gene alterations has been essential to select adequate treatments in the treatment of NSCLC ^12^.

Although NSCLC patients with *EGFR* active mutations respond to EGFR-TKIs, most of them will have disease progression with acquired resistance after a median of 12 months. The mechanisms of acquired resistance have been identified. The most prevalent resistance mutation was the Thr790Met (T790M) point mutation at exon 20, which was revealed in approximately half of the first- or second-generation EGFR-TKI-resistant patients. Osimertinib is a third-generation, irreversible EGFR-TKI that selectively inhibits not only *EGFR* active mutations but also T790M. The AURA 3 trial was a phase 3 trial to elucidate the efficacy of osimertinib compared with platinum doublet chemotherapy in patients with T790M-positive advanced NSCLC who had disease progression after first-line EGFR-TKI ^13^. This trial demonstrated that osimertinib has a significantly greater clinical effect than chemotherapy for T790M-positive NSCLC patients. Based on this result, osimertinib was approved for patients who were identified as having the T790M mutation. Osimertinib therapy as a first-line treatment showed a significant survival benefit in patients with NSCLC harboring 19 deletions and L858R compared with first-generation EGFR-TKIs, including gefitinib and erlotinib ^14^. In current clinical practice, most NSCLC patients with common *EGFR* mutations receive osimertinib as first-line therapy; however, some EGFR-mutated NSCLC patients are treated with first- or second-generation EGFR-TKIs with/without anti-vascular endothelial growth factor therapy, and these patients still need to receive T790M testing after a failure of first- or second-generation EGFR-TKIs.

In analyses for the T790M mutation, histological specimens from the relapsing sites need to be obtained using “rebiopsy”. However, there are some issues in performing rebiopsy, such as difficulty accessing the relapsing site and patient rejection because of invasive procedures^15^. Currently, the liquid biopsy method to identify the T790M mutation has been available to resolve these issues. However, the detection sensitivity of the T790M mutation using liquid biopsy is relatively low, and it is difficult to apply liquid biopsy methods to all resistant patients. In addition, there are limited data on the appropriateness of cytological specimens to test the T790M mutation using the cobas EGFR Mutation Test, version 2 if a suitable tissue specimen was not obtained. Therefore, we conducted this multicenter, prospective observational study to investigate the details of rebiopsy, including the implementation or success rate of rebiopsy and the concordance rate for T790M mutation detection between histological and cytological specimens in EGFR-TKI-resistant NSCLC patients.

## Results

### Characteristics of patients with acquired resistance

**Figure 1:**
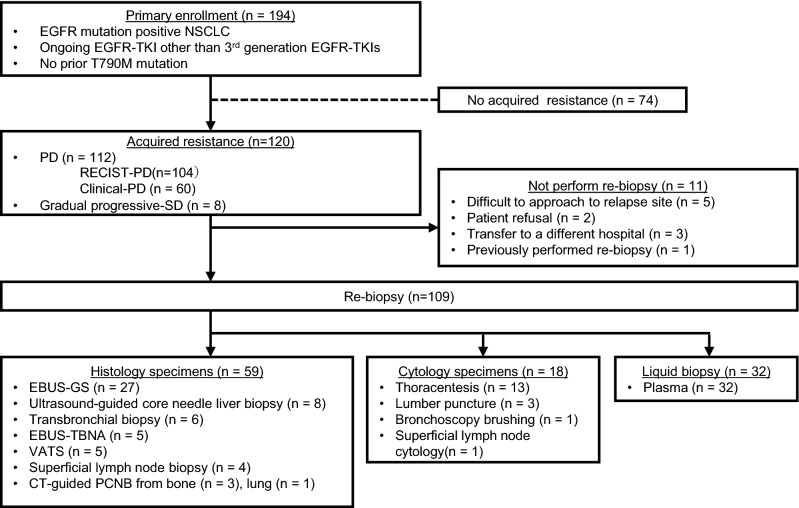
Overview of patient status for enrolled patients. One hundred ninety-four patients were enrolled in this study. One hundred twenty patients were defined as having acquired resistance to EGFR-TKIs, and 109 patients underwent rebiopsy.

A total of 194 patients were enrolled from February 2017 to January 2019. Among these, 120 patients were diagnosed with acquired resistance to ongoing EGFR-TKI therapy and were considered for undergoing rebiopsy (Fig. 1). The characteristics of patients who received rebiopsy are shown in Table 1. The types of acquired resistance were as follows: 104 patients (86.7%) had RECIST-PD, 60 (50%) had clinical PD, and 8 (6.7%) had SD with enlarged tumors. The median age was 68 years (range, 20–87), and 52.5% of the patients were women. Adenocarcinoma (99.2%) was the most common histology. Sixty-six patients (55%) had exon 19 deletions, 45 (37.5%) had L858R point mutations, and 10 (8.4%) had uncommon mutations, including G719X, L861Q and compound mutations. Forty-seven patients (39.2%) had been treated with erlotinib, 36 patients (30%) received gefitinib, and 37 patients (30.8%) had been treated with afatinib at disease progression. The median time from the start of EGFR-TKIs to rebiopsy was 14 months (range, 1.9 − 90 months).

**Table 1:** Patient characteristics in patients with acquired resistance (n = 120).

		n	(%)
Median age (range)		68	(20–87)
Gender	Male	57	(47.5)
	Female	63	(52.5)
Smoking history	Nonsmoker	73	(60.8)
	Smoker	47	(39.2)
Stage	IV	73	(60.8)
	Recurrence after surgery	40	(33.3)
	III	7	(5.8)
Histology	Adenocarcinoma	119	(99.2)
	Other	1	(0.8)
ECOG-PS	0	24	(20)
	1	79	(65.8)
	2	12	(10)
	3	4	(3.3)
	4	1	(0.8)
*EGFR* mutation	19del	66	(55)
	L858R	45	(37.5)
	Others	10	(8.4)
Prior EGFR-TKIs	Gefitinib	36	(30)
	Erlotinib	47	(39.2)
	Afatinib	37	(30.8)
Median treatment period of prior EGFR-TKIs (months) (range)		14	(1.9–90)
Reasons for acquired resistance	PD	112	(93.3)
	RECIST PD	104	(86.7)
	Clinical PD	60	(50)
	SD	8	(6.7)

*ECOG-PS*, Eastern Cooperative Oncology Group performance status; *EGFR*, epidermal growth factor receptor; *19del*, 19 deletions; *TKI*, tyrosine-kinase inhibitor; *PD*, progressive disease; *SD*, stable disease; *RECIST*, Response Evaluation Criteria in Solid Tumors.

### Results of rebiopsy

In 120 patients with acquired resistance to EGFR-TKIs, rebiopsy was performed on 109 patients, with an implementation rate of 90.8%, while 11 patients (9.2%) did not receive rebiopsy (Fig. 1). Reasons for not performing rebiopsy were as follows: difficulty accessing relapse sites (n = 5), patient refusal (n = 2), transfer to a different hospital (n = 3) and previously performed rebiopsy (n = 1).

Tissue histological specimens were obtained from 59 out of 109 patients (54.1%), including 27 out of 59 who received (45.8%) transbronchial biopsy using endobronchial ultrasonography with a guide-sheath (EBUS-GS); 8 (13.6%) with ultrasound-guided core needle liver biopsy; 6 (10.2%) with transbronchial biopsy without EBUS-GS; 5 (8.5%) with endobronchial ultrasonography-guided transbronchial needle aspiration (EBUS-TBNA); 5 (8.5%) with video-assisted thoracic surgery (VATS); 4 (6.8%) with superficial lymph node biopsy; 3 (5.1%) with bone biopsy using computed tomography (CT)-guided percutaneous core needle biopsy (PCNB); and 1 (1.8%) with lung biopsy using CT-guided PCNB. A summary of the rebiopsy results is shown in Table 2. In 59 patients, tissue histological specimens were diagnosed as follows: adenocarcinoma (n = 53, 90%) and transformation to other histology (n = 3, 5.1%). Two were small cell lung carcinomas, and 1 was a sarcomatoid cancer. No malignant tissue was collected by bone biopsy, EBUS-GS, or VATS in 3 patients. No PCR results were available from 8 patients. The success rate of rebiopsy using histological procedures was 81.3% (48/59). The T790M mutation was identified in 32 (54.2%) out of 53 patients with adenocarcinoma.

**Table 2:** Summary of re-biopsy result (n = 109).

	Diagnosis	n	(%)	T790M	n	(%)	The success rate of re-biopsy (%)
Histology specimens (n = 59)	Adenocarcinoma	53	(89.8)	Positive	32	(54.2)	81.3
				Negative	16	(27.1)	
				No PCR results	5	(8.5)	
	SCLC transformation	2	(3.4)				
	Sarcomatoid transformation	1	(1.7)				
	No malignant cells	3	(5.1)				
Cytology specimens (n = 18)	Adenocarcinoma/malignant cells	14	(77.8)	Positive	1	(5.6)	66.7
				Negative	11	(61.1)	
				No PCR results	2	(11.1)	
	SCLC transformation	1	(5.6)				
	No malignant cells	3	(16.6)				
Plasma liquid biopsy (n = 32)	Detection of any *EGFR* mutations	14	(43.8)	Positive	8	(25)	43.8
				Negative	6	(18.8)	
	No detection of *EGFR* mutations	18	(56.2)				
Total (n = 109)	Adenocarcinoma/malignant cells	81	(74.3)	Positive	41	(37.6)	67.9
				Negative	33	(30.8)	
				No PCR results	7	(6.4)	
	Transformation	4	(3.7)				
	No malignant cells	24	(22)				

*SCLC*, small cell lung cancer; *PCR*, Polymerase chain reaction; *EGFR*, epidermal growth factor receptor.

Only cytological specimens were available in 18 out of 109 patients (16.5%), including 13 (72.2%) thoracentesis; 3 (18.8%) lumbar puncture; 1 (5.6%) bronchoscopy brushing; and 1 (5.6%) superficial lymph node biopsy. In cytological specimens obtained from these 18 patients, malignant cells were revealed in 15 cytological specimens (83.3%). From cytological specimens, the T790M mutation was identified in only 1 patient (5.6%). No malignant cells were collected by thoracentesis or lumbar puncture in 3 patients. No PCR results were available from 6 patients. The success rate of rebiopsy using cytological procedures was 66.7% (12/18).

After the approval of plasma liquid biopsy using the cobas EGFR Mutation Test, version 2 for T790M detection in July 2017, 32 patients received liquid biopsy. In these 32 patients, the T790M mutation was identified in 8 patients (25%) and not identified in 6 patients (18.8%). Information on *EGFR* mutations, including T790M, 19 deletions, L858R, and uncommon mutations, was not obtained from liquid biopsy due to poor PCR products in 18 patients (56.2%). Thus, the success rate of liquid biopsy was 43.8% (14/32).

In total, the T790M mutation was identified in 41 patients (37.6%) out of 109 patients who received initial rebiopsy. The total success rate of rebiopsy was 67.9% (74/109). Additional rebiopsy after negative initial rebiopsy results was performed in 7 patients. Among the 7 patients who underwent a second rebiopsy, the T790M mutation was revealed in 2 patients (28.6%) by the cytology procedures. Finally, the T790M mutation was identified in 43 patients (43/109, 39.4%) in this observational cohort, including first and second rebiopsies. Problematic complications regarding rebiopsy were not reported.

### The concordance rate for T790M mutation detection between histological and cytological specimens

Both the histological and cytological specimens were obtained at the same time for the T790M mutation testing in 22 patients (Table 3). Each group had one patient who had a discrepancy for the T790M mutation result. Both the positive percent agreement and the negative percent agreement were 90.1% (10/11 for both), and the concordance rate for T790M mutation detection between histological and cytological specimens was 90.1% (20/22).

**Table 3:** The concordance rate for the T790M mutation detection between histology and cytology specimens (n = 22). The positive agreement; 90.9% (10/11), the negative agreement; 90.9% (10/11), the concordance rate; 90.9% (20/22).

	Cytology		
	Positive	Negative	Total
**Histology**			
Positive	10	1	11
Negative	1	10	11
Total	11	11	22

## Discussion

The importance of rebiopsy has increased in patients with *EGFR* mutations since the report of the AURA3 study results, which demonstrated the efficacy of osimertinib for patients with T790M-positive NSCLC. It is suggested that the implementation rate of rebiopsy may be associated with the prognosis of EGFR-TKI-refractory NSCLC patients ^16^. In the current observational study, the implementation rate of rebiopsy was 93.4% (109 out of 120 patients with acquired resistance), with a T790M-positive rate of 39.4% (43 out of 109 patients receiving rebiopsy). To our knowledge, the implementation rate of rebiopsy was the highest compared with previous retrospective or observational studies investigating T790M detection in *EGFR*-mutated NSCLC patients after EGFR-TKIs. The implementation rate of rebiopsy has been reported to range from 55.1 − 63% in retrospective studies and 86.9% in the Japanese observational study named the REMEDY ^15,17,18^. The use of liquid biopsy might obviously affect the high implementation rate of rebiopsy. Liquid biopsy has significant advantages of feasibility, such as noninvasiveness and accessibility, for detecting the T790M mutation ^19^. Indeed, the most common procedure of rebiopsy in the REMEDY study was liquid biopsy using plasma, accounting for 58.1% ^17^. In the current study, 28.9% of patients received liquid biopsy as a rebiopsy procedure. However, liquid biopsy for EGFR-TKI-resistant patients has some limitations. The first problem is the transformation to other histopathological features, such as small cell lung carcinoma (SCLC) transformation, which cannot be detected by liquid biopsy ^20^. There were four transformation cases (3 small cell lung carcinoma and one sarcomatoid cancer) in this study. The efficacy of osimertinib may be limited for the concomitant case of the T790M mutation and SCLC transformation ^21^. The second problem of liquid biopsy using plasma is the lower detection rate of the T790M mutation. A previous study demonstrated that one-third of patients who underwent liquid biopsy had the T790M mutation according to the cobas EGFR Mutation Test ^22^. In this study, the success rate of liquid biopsy was 43.8%, and the detection rate of the T790M mutation was 25%. These results indicated that liquid biopsy could be a useful option for patients for whom tissue samples were not available.

The success of rebiopsy is important to determine the subsequent treatments for EGFR-TKI-refractory patients. In this study, the success rate of the histological procedure (81.3%) was higher than that of the cytological procedure (66.7%) or liquid biopsy (43.8%). The main reason for the selection of liquid biopsy as a rebiopsy procedure was the inaccessibility to relapse lesions, such as bone metastases, central nerve metastases or small lung metastases (data not shown). A skilled expert and adequate devices are needed to perform the invasive procedures accessing such relapse lesions, which may depend on the status of participating institutions. The rebiopsy sites and methods in histology-based procedures may affect the positive rate of the T790M mutation. In this study, the positive rates of the T790M mutation by rebiopsy sites, lung, lymph node, liver and bone, were 47.4% (18/38), 100% (10/10), 50% (5/10) and 66.7% (2/3), respectively. The positive rates of the T790M mutation by BF, open biopsy and needle biopsy were 47.4% (18/38), 77.8% (7/9) and 58.3% (7/12), respectively. It is important to cooperate with each institution in local communities to improve the success rate of rebiopsy.

The concordance of tissue or plasma samples was well investigated in a previous report ^23^. However, the data regarding the concordance of histological and cytological samples have been insufficient. Transbronchial procedures are common methods used to perform rebiopsy because of the availability and feasibility of bronchoscopy. The percentage of patients who received rebiopsy by transbronchial procedures without liquid biopsy was 50.6% (39/77), including EBUS-GS, transbronchial biopsy, EBUS-TBNA and BF brushing in this study (Fig. 1). The percentages of rebiopsy procedure transbronchial procedures in prospective cohort trials were 52.4% (43/82) and 63.9% (69/108) ^17,24^. However, it is difficult to obtain sufficient tissue samples for histology-based testing by transbronchial procedures from all patients. We investigated the accuracy of cytology-based T790M detection compared with histology-based testing using tissues obtained at the same time. As a result, the positive percent agreement and the negative percent agreement were both 90.1% (Table 3). DNA degradation by formalin in histology-based procedures and a small percentage of malignant cells in cytology-based procedures might result in these discordant mutation results. These results indicate that cytological specimens obtained by transbronchial procedures could be useful for detecting the T790M mutation.

This study has several limitations. First, when this study was conducted, liquid biopsy was not approved for use in clinical practice in Japan. The main limitation of this study was that the implementation rate of rebiopsy could not be evaluated according to the original statistical hypothesis because of the approval status of liquid biopsy in Japan. Indeed, 5 patients did not receive rebiopsy because of difficulty accessing relapse sites and the unavailability of liquid biopsy. Second, in this study, liquid biopsy was performed using the cobas EGFR Mutation Test, version 2, which can detect only *EGFR* mutations in exons 18–21. This restriction by PCR-based methods could affect the lower success rate of liquid biopsy. In current clinical practice, next-generation sequencing (NGS) panels, such as FoundationOne Liquid CDx and Guardant360 CDx, cover more mutations with higher sensitivity than the cobas EGFR Mutation Test, version 2. It is expected that a higher success rate of liquid biopsy can be achieved using the NGS panel. Future studies are warranted to compare the success rate of liquid biopsy by the NGS oncology panel with histology- and cytology-based procedures. In addition, the NGS panel could detect other mutations that cause acquired resistance to EGFR-TKIs. NGS-based testing will become the mainstream rebiopsy method to address this situation in the future. Third, information on treatment after rebiopsy was not collected in the present study. We did not include treatment after rebiopsy as an endpoint; therefore, this information is missing.

Revealing the resistance mechanisms and developing a strategy for overcoming resistance to EGFR-TKIs remain the main focuses of the investigation of *EGFR* mutation-positive patients. After the report of the FLAURA study, the importance of T790M detection has decreased because most physicians tend to choose osimertinib for chemo-naive *EGFR* mutation-positive NSCLC patients. However, new resistance mechanisms have been identified from the analysis using liquid biopsy after treatment with osimertinib in the FLAURA trial ^25^. The most frequent resistance mechanisms were MET amplification (15%) and secondary *EGFR* mutations (10%), including C797X and uncommon mutations. These resistance mechanisms will become new targets to overcome osimertinib resistance, and a number of clinical trials are ongoing in this area. Clinical investigations concerning rebiopsy procedures and mutation detection methods should be continued for future driver-based medicine. The current study demonstrated that rebiopsy was feasible and could provide useful data to select subsequent treatments even in clinical practice.

## Methods

### Study design

The present study was a multicenter, prospective, observational study in patients with *EGFR* mutation-positive advanced NSCLC who experienced disease progression during treatment with EGFR-TKIs. Patients with advanced NSCLC meeting all inclusion criteria and not meeting exclusion criteria were consecutively enrolled from medical institutions participating in the Niigata Lung Cancer Treatment Group (NLCTG) to collect information on rebiopsy data. This study was conducted according to the principles of the Declaration of Helsinki. The institutional review board of each participating institution (Niigata Cancer Center Hospital, Niigata University Graduate School of Medical and Dental Sciences, Nagaoka Red Cross Hospital, Nagaoka Chuo General Hospital, Niigata Prefectural Central Hospital, Niigata City General Hospital, Saiseikai Niigata Hospital, Shibata Hospital—Niigata Prefectural Hospital, Nishi Niigata Chuo Hospital, Sado General Hospital, Tsuruoka Municipal Shonai Hospital, Shinrakuen Hospital, Niigata Medical Center) approved the protocol. This study is registered in February 2017 on the clinical trials site of the University Hospital Medical Information Network Clinical Trials Registry (registration number: UMIN000026019). Information on patient demographics was collected retrospectively from the medical records at study enrollment. Additional data regarding rebiopsy were collected when the following situations were met: sustained response to EGFR-TKI (compete response, pertain response, or stable disease more than 6 weeks) and acquired resistance to ongoing EGFR-TKI. The definition of acquired resistance in this study was as follows: progressive disease according to Response Evaluation Criteria in Solid Tumors (RECIST) criteria (RECIST-PD), clinically progressive disease assessed by physicians (clinical PD), and stable disease according to RECIST criteria but increasing tumor volume compared with baseline (SD with enlarged tumor). The collected data were as follows: patient status at the point of rebiopsy, information on rebiopsy sites (relapse site, size, number, and so on) and the rebiopsy procedure, the results of rebiopsy (cytology, histology, and the T790M status), complications regarding rebiopsy, subsequent treatments, and prognosis. If patients did not undergo rebiopsy at the point when acquired resistance developed, the reasons for not undergoing rebiopsy, including patient refusal, were collected. The existence of malignant cells was determined by pathologists at each participating institution. All data were collected using electronic data capture.

### Patients

The inclusion criteria were as follows: a histologically or cytologically confirmed advanced NSCLC with *EGFR* active mutations; stage III/IV not amenable to definitive radiation therapy or postoperative recurrence at the start of EGFR-TKI therapy; ongoing EGFR-TKI therapy (gefitinib, erlotinib or afatinib); no prior T790M mutation detection; written informed consent for study participation from the patient. Patients treated with third-generation EGFR-TKIs were excluded. Prior cytotoxic chemotherapies were allowed. Written informed consent was obtained from all study participants.

### Study endpoints

The primary endpoint was the implementation rate of rebiopsy, defined as the number of patients who underwent rebiopsy from any site/total number of patients with acquired resistance to EGFR-TKIs. After approval of liquid biopsy testing for the T790M mutation in July 2017, liquid biopsy, which was performed using the cobas EGFR Mutation Test, version 2, was also counted as rebiopsy. The coprimary endpoint was the concordance rate for T790M mutation detection between histological and cytological specimens using the cobas EGFR Mutation Test, version 2. The concordance rate for T790M mutation detection between histological and cytological specimens was defined as the percentage of patients with T790M positivity according to cytological specimens among patients with T790M positivity according to histological specimens. Cell block analysis was included in cytological specimens. Secondary endpoints were as follows: the success rate of rebiopsy according to procedure, the T790M mutation-positive rate according to biopsy site or procedure, safety of rebiopsy procedures, reasons for patients not undergoing rebiopsy, and implementation rate of rebiopsy according to institution and available rebiopsy procedures. For histology- and cytology-based procedures, the success rate of rebiopsy was defined as the percentage of patients in whom malignant cells and the results of gene mutation analysis were available among the patients who underwent rebiopsy. For liquid biopsy, the success rate of rebiopsy was defined as the percentage of patients in whom EGFR mutations were detected among the patients who underwent liquid biopsy.

### Statistical analyses

In a retrospective study regarding rebiopsy, the implementation rate of rebiopsy was reported to be 62.5%, and the percentage of patients who could be examined for *EGFR* gene mutations using histological specimens was reported to be 28% ^16^. We estimated the lower limit to be able to examine the T790M mutation in both histological and cytological specimens to be 30%. If we assumed a threshold rate of 0.5 and an expected rate of 0.8, 83 patients were needed to evaluate the positive percent agreement of T790M mutation detection between histological and cytological specimens with 80% power and an alpha level of 0.05 (2-sided). Even if a dropout rate of approximately 5% was considered, it was estimated that 100 patients was the target number of patients who had acquired resistance. This sample size would have a half-width of the exact 95% confidence interval of ± 10% or less for the implementation rate of rebiopsy. SAS version 9.4 (SAS Inc., Cary, NC, USA) was used for the sample size evaluation.
